# Movement distributions of stroke survivors exhibit distinct patterns that evolve with training

**DOI:** 10.1186/s12984-016-0132-y

**Published:** 2016-03-09

**Authors:** Felix C. Huang, James L. Patton

**Affiliations:** Sensory Motor Performance Program, Rehabilitation Institute of Chicago, 345 E, Superior Street, Suite 1406, Chicago, IL 60611 USA; Department of Bioengineering, University of Illinois at Chicago, 851 S. Morgan Street, Room 222, Chicago, IL 60612 USA

**Keywords:** Robotic rehabilitation, Upper extremity, Customization

## Abstract

**Background:**

While clinical assessments provide tools for characterizing abilities in motor-impaired individuals, concerns remain over their repeatability and reliability. Typical robot-assisted training studies focus on repetition of prescribed actions, yet such movement data provides an incomplete account of abnormal patterns of coordination. Recent studies have shown positive effects from self-directed movement, yet such a training paradigm leads to challenges in how to quantify and interpret performance.

**Methods:**

With data from chronic stroke survivors (*n* = 10, practicing for 3 days), we tabulated histograms of the displacement, velocity, and acceleration for planar motion, and examined whether modeling of distributions could reveal changes in available movement patterns. We contrasted these results with scalar measures of the range of motion. We performed linear discriminant analysis (LDA) classification with selected histogram features to compare predictions versus actual subject identifiers. As a basis of comparison, we also present an age-matched control group of healthy individuals (*n* = 10, practicing for 1 day).

**Results:**

Analysis of range of motion did not show improvement from self-directed movement training for the stroke survivors in this study. However, examination of distributions indicated that increased multivariate normal components were needed to accurately model the patterns of movement after training. Stroke survivors generally exhibited more complex distributions of motor exploration compared to the age-matched control group. Classification using linear discriminant analysis revealed that movement patterns were identifiable by individual. Individuals in the control group were more difficult to identify using classification methods, consistent with the idea that motor deficits contribute significantly to unique movement signatures.

**Conclusions:**

Distribution analysis revealed individual patterns of abnormal coordination in stroke survivors and changes in these patterns with training. These findings were not apparent from scalar metrics that simply summarized properties of motor exploration. Our results suggest new methods for characterizing motor capabilities, and could provide the basis for powerful tools for designing customized therapy.

## Background

Interactive technologies have shown some success in improving upper extremity function [1–4], yet the best practices for effective therapy have remained unclear. One key limitation in many approaches is the failure to account for differences in impairments across individuals. In stroke survivors, for example, these can include loss of sensation, spasticity, imbalanced muscle strength [5, 6], co-contraction [7], abnormal muscle coupling [8], with each at varying levels of severity [9–11]. Clinical assessments provide tools for characterizing individual motor abilities [12, 13], yet concerns remain over their repeatability and reliability. Recent work with interactive robotic and visual feedback systems has focused on capabilities unique to such technology: repeatable and objective measurement of motor behavior at high spatial and temporal resolution [14–16]. However, while such measurements allow for great precision, it is not immediately clear how they can provide a greater understanding of motor impairments.

One potential approach to characterizing impairment is to examine trends found from data recording devices during training. For example, movements following stroke often exhibit limits to range of motion, or incoordination between joints [17]. Abnormal muscle tone or coupling [8] can often manifest as stereotyped gestures. Beyond errors in goal-directed movement, motor impairment results in general changes in coordination patterns. While historic approaches in robot-assisted therapy began with guidance [18, 19] during repeated reaching tasks, such repetition focuses only on the iterative tuning of a particular motor plan. Prescribed task goals might then impose constraints that obscure how motor impairments would naturally manifest.

An obvious next step is to characterize freeform *motor exploration*, or patient-directed movement practice [20, 21]. Variability in the degrees of freedom outside of the task-space has been found to be critical to optimizing task goals [22, 23]. Yet exploratory movements are themselves thought to facilitate infant motor development [24, 25], while task variety benefits learning generalization [26]. There is a large body of literature that describes variability as a sign of health while invariant behavior is a sign of pathology [27–31]. In terms of rehabilitation, self-directed practice has been used as a control condition to examine the clinical outcomes of new interventions [32, 33], for which instrumented recording was not involved. We argue that the variable and sustained nature of exploratory movement has advantages for characterizing impairment. Beyond information about the average or extent of capabilities, motor exploration could uncover whether movement gestures are over- or under-expressed.

To better understand motor impairment within such a movement paradigm, we believe that multivariate statistical analyses should be employed. Recently, researchers examined free motor expression using principal components analysis as the basis for describing available movement in the shoulders and torso for individuals with spinal cord injury [34, 35] and for re-mapping finger movements to screen coordinates [36]. Gizster and Mussa-Ivaldi showed that a vector field could be used to summarize the tendencies of spinal motor circuits in generating forces across space [37, 38]. Recently, Pataky and colleagues employed multivariate statistics to objectively analyze multi-dimensional kinematic and kinetic trajectories using statistical parametric mapping (SPM) techniques [39, 40]. Beyond capturing overall statistical features, we undertake to model the entire distribution of movement to reveal a more comprehensive view of a patient’s particular deficits in movement tendencies.

This study investigated how patterns of movement within motor exploration evolve during training and to what extent they differ between individuals. Our recent work showed that motor exploration combined with negative viscosity from a robotic interface improved learning in healthy individuals and in stroke survivors [21, 41]. Here, we considered new analyses on data from our previous work with stroke survivors. Focusing on the control condition in which no external forces were applied, we examined how the statistical distribution of kinematic variables (displacement, velocity, acceleration) changed over the course of training. In addition, we performed a new data collection with healthy individuals to serve as a control group. One possible outcome is that motor deficits manifest as highly variable movements, such that no systematic patterns can be found. Alternatively, distributions potentially could reveal stereotyped patterns that correspond to an individual’s unique form of motor impairment and recovery.

## Methods

### Experiment participants

We consider data from a previous study in which stroke survivors performed manual exercises using a planar robotic device. Stroke survivors performed the task with their affected arm. Note that upper extremity Fugl-Meyer scores and lesion data were available for only some individuals from previously obtained assessments and hospital records. We have assigned each stroke survivor an identification code (A-J), which was then associated with the results of each individual for clinical data (see Table 1), as well as the distribution analyses proposed in this study. Individuals in this experiment group were paid for their participation. We collected data for 10 healthy individuals to serve as an age-matched control group (mean age: 52.0 ± 11.0 SD). This group practiced for a single session using the same protocol, but with their dominant arm only. Individuals in the control group were not paid for their participation. Each individual provided informed consent in accordance with the Northwestern University Institutional Review Boards, which specifically approved this study and follows the principles expressed in the Declaration of Helsinki.

**Table 1:** Stroke survivor lesion and clinical assessment data

Subject ID	Age	Handedness	Side of paresis	Months post stroke	Stroke type	UEFM	Stroke location
A	47	N/A	L	37	N/A	N/A	N/A
B	47	L	R	6	ischemic	45	Subcortical
C	55	R	L	265	N/A	19	Subcortical
D	67	R	R	67	ischemic	N/A	MS, Brainstem and cortical
E	60	R	L	28	ischemic	44	Subcortical and Cortical
F	55	R	L	24	ischemic	11	Subcortical and Cerebellar
G	41	R	L	51	ischemic	25	Subcortical
H	57	R	R	131	ischemic	59	Cortical
I	58	N/A	L	20	ischemic	N/A	Subcortical and Cortical
J	41	R	L	138	ischemic	23	Cortical

### Experiment protocol

**Fig. 1:**
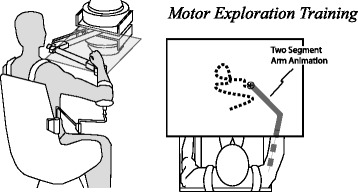
Experiment Apparatus. The robotic device interfaced to the arm about a free pivot at the wrist. Experiment participants performed self-directed motor practice. This study focused on the distribution of the observed movement in the absence of robot forces

We asked experiment participants to control the movement of a planar force-feedback device as described in our previous work [42]. To focus training on the coordination of the forearm and upper arm, each participant operated the device through a brace, pivoted at the wrist. Using an overhead projector mounted on the ceiling, real-time feedback of the handle position, visual reference cues, and experiment instructions were presented on a horizontal surface overlaying the planar workspace of the arm (see Fig. 1). In addition, the real-time animation included two segments approximating the motion of the forearm and upper arm. Visual reference cues included a larger rectangular region, indicating the bounds of movement for the motor exploration portions of the experiment.

For the motor exploration task, we instructed participants to move the handle using a variety of directions, speeds, and positions within the rectangular workspace (0.2 x 0.6m). We explained that each exploration phase should serve as preparation for a subsequent evaluation phase in which participants would perform movements around a circular track of 0.1 m radius. To control for the level of training exposure between participants, the computer provided a cue to halt motor exploration after 25 m of handle endpoint travel. This target was selected simply to provide some task variety between exploration and goal-directed movement phases of the experiment. Each session included several alternating exploration phases (16) and evaluation trials in which participants performed the circular movements (160). Only the exploration phases were analyzed for the current investigation. Distance rather than time duration was chosen as a control so that the trials could only progress through active involvement. Stroke survivors performed only three sessions on different days, typically completed within a 7-day period. Each session was divided into two 1-h blocks, with a 15-min intervening break. The stroke survivors in this study did not receive any other therapy during their participation in this study. Note that the healthy individuals involved in this additional data collection only participated for a single session.

### Data analysis

Analysis of distribution model components: As our primary analysis for this study, we examined the distributions of displacement, velocity and acceleration for planar motion, as expressed by the stroke survivors and healthy individuals during motor exploration. We examined whether modeling analysis of distributions could reveal changes in available movement patterns. We desired a modeling framework that could describe multiple clusters of data within a given dimension, each with a different mean center and variance. We fitted recorded histograms with a weighted sum of multivariate normal (Gaussian) distributions according to maximum likelihood estimates:

Eq-1 $$ {\boldsymbol{f}}_{\boldsymbol{x}}\left({x}_1,\dots,\ {x}_k\right)={\displaystyle \sum_1^J\frac{1}{{\left(2\pi \right)}^{\frac{k}{2}}{\left|S\right|}^{\frac{1}{2}}} \exp \left(-\frac{1}{2}{\left(\boldsymbol{x}-{\boldsymbol{\mu}}_{\boldsymbol{j}}\right)}^T{S}^{-1}\left(\boldsymbol{x}-{\boldsymbol{\mu}}_{\boldsymbol{j}}\right)\right)} $$

for *k* dimensions (for example x and y axes of displacement), and *j* number of model components. Increasing *j* improves model fitness by adding a single Gaussian function centered at *μ*_*j*_ covariance described by a matrix *S*_*j*_, corresponding to a cluster of data. We fit this model to the observed hand motion distributions for each exploration trial, resulting in two-dimensional histograms for displacement, velocity and acceleration were normalized so that sum of observations was unity. We evaluated model fitness according to the coefficient of determination, R^2^. The change in model fitness relative to the number of model components was examined over the course of training sessions. Considering models with 1 to 10 components, we summarized the results in terms of the change between the first and last day of training (Day 1 and Day 3). We employed paired T-tests to evaluate significant differences in the coefficient of determination due to increasing model components.

#### Scalar analysis of motor exploration

To better understand the potential novel contribution of distribution analysis we compared our results with scalar measures of performance. To this end, we propose some established as well as novel methods that we believe will be suitable to characterize distributed data in human movement. Here we focused on how measures of the range of motion change from day to day for displacement, velocity and acceleration. These choices served as simple scalar counterparts to the distribution analyses in this paper. We defined the *range* as the span between the 25^th^ to 75^th^ percentiles (inter-quartile) of observed data. We computed the workspace area (displacement data), and analogously for velocity and acceleration, as the *products inter-quartile range* for the two axes of motion (left-right and fore-aft, defined as *x* and *y* degrees of freedom). This measure was chosen to capture the typical movement, while being less sensitive to rare occurrences at the extreme limits of motion.

Beyond the range of motion, we also considered whether standard statistical measures [43, 44] of uniformity can reveal changes in motor exploration: joint entropy and covariance in the planar motion. Finally, an important consideration is how the behavior during motor exploration could provide information about motor capability. While this issue is difficult to address comprehensively, we examined correlations between metrics of motor exploration versus subsequent performance in circular motion tasks using some established metrics of human movement (including mean radial deviation [21], movement variability, speed variability). We also included a new metric ‘frequency mismatch’ that we define as the difference between the average movement frequencies between the lateral and fore-aft directions of movement. This metric quantifies the error of coordination between degrees of freedom during execution of circular motion. Such correlations could reveal how scalar metrics of motor exploration predicts performance in goal directed actions. We employed paired T-tests to evaluate significant change of metrics between training sessions. A two-tailed probability value of 0.05 was used for statistical significance criterion in all analyses.

#### Individual differences

To determine whether individuals’ patterns of motor exploration could be uniquely identified, we performed classification analyses in which histogram count values served as candidate features for classification. The classifier algorithm was “trained” by associating the pattern histogram counts at certain states (e.g. a particular velocity) with each experiment participant. The classifier is then “tested”, by evaluating its predictive power for data that was not considered in the original analysis. Using only data from the first session, training and test sets were constructed from alternating trials. A reduced set of features were obtained from histogram bins that were significantly different than the group mean containing over 0.5 % of data. We then performed linear discriminant analysis (LDA) classification with the selected features, using ‘classify’ function with MATLAB software (MATHWORKS, Natick, MA), to compare predictions versus actual subject identifiers.

To characterize the performance of the classifier, we computed the overall success rate for identification of individual test trials. This measure of classification accuracy reflects the success of identification if we only relied on a single trial of motor exploration data. We also summarized the classifier performance in terms of a measure of sensitivity: S = TP/ (TP + FN), where TP (true positive) is the number of instances for correctly identifying an individual’s trial data, and FN (false negative) is the number of instances of incorrectly rejecting a trial. This latter measure is a more practical measure of the degree the classifier shows that individuals are distinct. The data was separated into training versus test sets in a 50-50 % ratio.

## Results

**Fig. 2:**
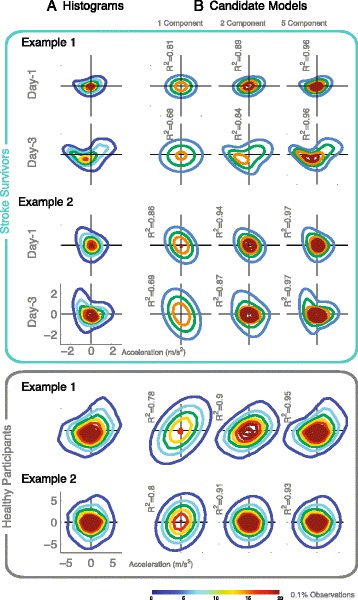
Distribution Model Component Analysis. **a** Contour plots of acceleration histograms (shown for two typical stroke survivors, top) versus multivariate normal functions **b** with 1, 2, and 5 components, reveal new movement patterns from Day-1 and Day-3 (Red/blue indicates greater/lesser observations). Age-matched control participants (shown for two typical individuals, bottom) practiced for one session, exhibiting distributions with simpler structure and wider overall range

**Fig. 3:**
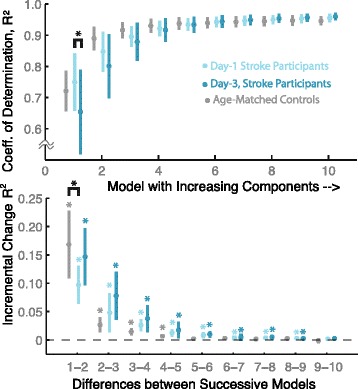
Comparison of Groups and Effect of Training. The coefficient of determination R^2^ indicated the goodness of fit (95 % CI across subjects) of multivariate normal functions to observed histograms of kinematic variables (acceleration shown) for 10 stroke survivors (blue) and 10 age-matched control participants (grey). With one model component, Day-3 (dark blue) exhibited worse fit (*p* = 0.008, paired *t*-test, upper plot) compared to Day-1 (light blue). Increasing components (lower plot) yielded incrementally improved fit with stroke survivors compared to the control group (up to 9 versus 5 components). These trends suggest that stroke survivors exhibited overall more complex distributions of acceleration, and that extended practice increased that complexity

The goal of this study was to determine how analysis of motor exploration could be used to characterize movement capability in stroke survivors. Our first key finding was that extended practice resulted greater complexity in the resulting movement distributions. Acceleration data fit to multivariate normal functions revealed that additional model components were needed to accurately represent the last day of training. A summary analysis for all subjects (see Fig. 2) showed that coefficients of determination (R^2^) values were lower (mean change: -0.112 ± -0.1043) for Day-3 (mean: 0.64 ± 0.18) distributions compared to Day-1 (mean: 0.75 ± 0.13) when considering a single component (*p* = 0.008, paired *t*-test). For one component, the age-matched control group exhibited a comparable average R^2^ as the stroke survivors in this study (see Fig 3). This similarity could reflect a common feature of repeated observations near zero velocity. While the current presentation focuses on acceleration, note that analyses of velocity and displacement distributions revealed similar trends of more model components needed for Day-3 compared to Day-1.

While use of a single component model provides a convenient reference point for revealing change, our analysis of increasing model components reveals evidence that stroke survivors have relatively complex movement distributions. The healthy individuals in this study exhibited distributions of acceleration that were more alike, with simpler structure (see Fig. 2). For all distributions, increasing the number of Gaussian elements had the effect of improving the goodness of fit, with clear diminishing returns with each additional component. However, we found differences that suggest that increasing components has more utility in the data from stroke survivors.

To illustrate this, let us consider the impact of increasing model components in fitting acceleration data. We found that the increase in the coefficient of determination (see Fig. 3) from one to two components was greater (mean difference: 0.073, CI: 0.0074, 0.0138; *p* = 0.031) for healthy individuals (mean 0.168, CI: 0.108, 0.228) compared to stroke survivors (mean 0.096; CI: 0.059, 0.132). In addition, incrementally increases up to five components resulted in improved R^2^ (mean change: 0.00127; CI: 0.0040, 0.0094; *p* = 3.06e-4), while increases up to nine elements resulted in improved R^2^ (mean change: 0.00300; CI: 0.0013, 0.0048; *p* = 0.0038) for stroke survivors (first day).

**Fig. 4:**
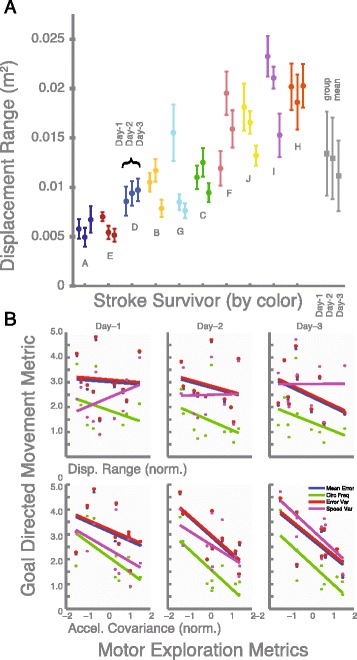
Scalar Summary Metrics. **a** Stroke survivors did not as a group change in the range of motion during motor exploration training, according to analyses of the inter-quartile product for displacement. However, analysis of data by individual (coded by color, sorted by individual mean position range, shown with 95 % CI across trials as error bars) and between days (each triplet) did in fact reveal cases of both significant increased and decreased range. Similar results were found for velocity and acceleration. **b** Linear regressions between motor exploration metrics and performance in subsequent goal directed actions (circular movements) did not in general reveal significant trends. However, the covariance of acceleration appeared to correlate with several metrics of goal directed movements, which suggests that the distribution of acceleration is an important feature describing motor ability

To provide better context for the insights gained from analysis of motor exploration, an important consideration is its relationships to traditional metrics (see Fig. 4). Using linear regression, we found that metrics of motor exploration (i.e. the range of displacement, velocity, and acceleration) largely did not correlate with typical performance metrics in subsequent tests of performing circular movements, for example, average error, movement smoothness, speed variability (see Table 2). We did find some indications of significant correlation between the covariance of acceleration during motor exploration versus each of the tested metrics for circular movement (Day-2; *p* = 0.011, 0.0077, 0.013. 0.11; Day-3: *p* = 0.027, 0.078, 0.028, 0.0073). It should be noted, however, that the magnitudes of *p*-values observed here indicate that there is a strong possibility of finding accidental trends given even the modest number of metrics considered. Nonetheless, these analyses suggest that focusing on the distribution patterns of acceleration will be more fruitful for determining a relationship between motor exploration and goal-directed movement. While it is possible to summarize important features of motor exploration data as a scalar metric, we argue that distribution analysis is more useful because it highlights the inherent multivariate nature of such data.

**Table 2:** Correlation of motor exploration and goal-directed movement

	Day-1	Day-2	Day-3
Motor Exploration Metric: Displacement Range (m) ^2^			
i	−0.046 CI:-0.618, 0.527	−0.175 CI:-0.778, 0.427	−0.380 CI:-1.087, 0.328
ii	−26.3 CI:-95.5, 42.9	−42.4 CI:-130.4, 45.6	−44.2 CI:-145.5 57.2
iii	−0.063 CI:-0.757, 0.631	−0.221 CI:-0.952, 0.510	−0.457 CI:-1.305, 0.391
iv	2.97 CI:-3.35, 9.29	0.18 CI:-5.94, 6.30	0.040 CI:-5.623, 5.704
Motor Exploration Metric: Velocity Range (m/s) ^2^			
i	0.000 CI:-0.026, 0.026	−0.002 CI:-0.035, 0.031	−0.012 CI:-0.046, 0.022
ii	−0.077 CI:-3.337, 3.183	−1.541 CI:-6.371, 3.289	−1.131 CI:-5.924, 3.662
iii	−0.000 CI:-0.032, 0.031	−0.003 CI:-0.043, 0.036	−0.015 CI:-0.055, 0.026
iv	**0.241 CI:0.007, 0.474**	0.091 CI:-0.223, 0.406	0.068 CI:-0.183, 0.319
Motor Exploration Metric: Acceleration Range (m/s^2^) ^2^			
i	0.000 CI:-0.001, 0.001	0.001 CI:-0.001, 0.003	0.000 CI:-0.002, 0.002
ii	0.021 CI:-0.122, 0.164	0.043 CI:-0.284, 0.370	0.052 CI:-0.212, 0.315
iii	0.000 CI:-0.001, 0.001	0.001 CI:-0.001, 0.004	0.000 CI:-0.002, 0.002
iv	**0.011 CI:0.000, 0.021**	0.013 CI:-0.005, 0.032	0.008 CI:-0.004, 0.021
Motor Exploration Metric: Acceleration Covariance (m/s^2^)			
i	−0.004 CI:-0.013, 0.004	**−0.008 CI:-0.014, -0.002**	**−0.008 CI:-0.014, -0.001**
ii	−0.894 CI:-1.830, 0.042	**−1.257 CI:-2.077, -0.437**	**−1.197 CI:-1.979, -0.415**
iii	−0.005 CI:-0.016, 0.005	**−0.010 CI:-0.017, -0.003**	**−0.009 CI:-0.017, -0.001**
iv	−0.067 CI:-0.164, 0.029	−0.056 CI:-0.128, 0.016	**−0.063 CI:-0.104, -0.022**
Motor Exploration Metric: Joint Entropy			
i	−0.000 CI:-0.008, 0.008	0.003 CI:-0.008, 0.013	0.006 CI:-0.003, 0.016
ii	−0.160 CI:-1.190, 0.870	0.427 CI:-1.147, 2.001	0.719 CI:-0.640, 2.079
iii	−0.000 CI:-0.010, 0.010	0.003 CI:-0.009, 0.016	0.007 CI:-0.004, 0.019
iv	−0.065 CI:-0.146, 0.016	−0.017 CI:-0.121, 0.086	0.014 CI:-0.063, 0.091

Note 1: Values indicate linear regression coefficient *m* in *y = mx + b*

Note 2: Rows correspond to goal-directed movement metrics:

i. Mean Radial Deviation (m), ii. Frequency Mismatch (rad/s) iii. Error Variability (m) iv. Speed Variability (m/s)

Note 3: Bold text indicates *p* < 0.05

Our next major goal in this study was to determine the patterns of motor exploration differed between individual stroke survivors. Using a correlation analysis of movement histogram data, we tested how well 50 % of a participant’s data could predict the other half of their data, and compared this to how well this could predict other individuals. We found that the mean coefficient of determination for self-to-self comparisons was generally high (0.90 ± 0.05, 0.90 ± 0.07, 0.95 ± 0.03) while the self-to-others was low (0.18 ± 0.14, 0.21 0.17, 0.18 ± 0.23) for the displacement, velocity, and acceleration distribution analyses, respectively. These results demonstrate that a significant portion of distributions differed between individuals. As a reference for the reader, we present the histograms for all stroke survivors participants for displacement, velocity, and acceleration for all three days (see Fig. 6).

**Fig. 5:**
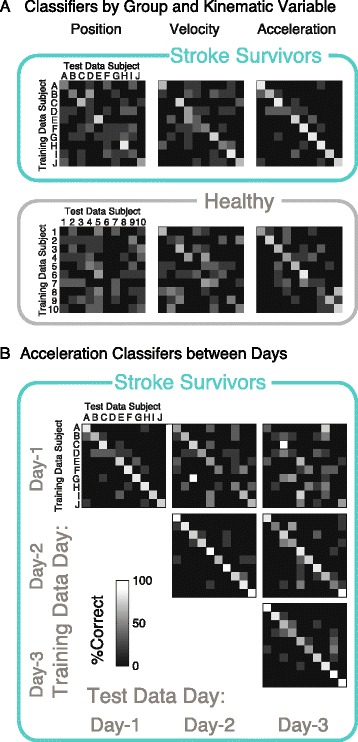
Individual Differences. **a** A classifier algorithm was “trained” with acceleration histogram data to identify a test data set (50-50 training versus test) for displacement, velocity, and acceleration (columns), separately for stroke survivors (*n* = 10, top) and aged-matched controls (*n* = 10, bottom). Clearer differences were found between stroke survivors using acceleration. **b** For stroke survivors, data from the same day (major diagonal elements) as opposed to different days (major off-diagonal elements) yielded better classification accuracy

Classification analysis served as a more direct measure of whether stroke survivors exhibited patterns that were distinct from each other. Interestingly, this analysis revealed more accurate classification for the higher derivatives of motion (i.e. acceleration versus velocity and displacement). Focusing on Day-1, the LDA-classifier identified subjects correctly for 75.0 % of the trials when using acceleration data, while it was 51.2 % and 41.2 % correct for velocity and displacement (Fig. 5). The overall sensitivity for the classifier was 0.98 for acceleration, 0.96 for velocity, and 0.88 for displacement, showing that each subject’s unique signature could be captured.

In contrast to the distribution analysis above, our examination of traditional scalar metrics did not reveal significant changes across days. For the group as a whole, day-to-day changes were not detected according to analysis of the products of inter-quartile range. The change in the observed range of motion from Day-1 and 3 was not significant in terms of the interval between the 25^th^ and 75^th^ percentiles (*p* = 0.13, 0.67, 0.87; product of inter-quartile change for *x*, lateral and *y*, fore-aft) for displacement (-0.0022 ± 0.00043 m^2^), velocity (0.0087 ± 0.062 (m/s)^2^), or acceleration (0.084 ± 1.55 (m/s/s)^2^). We found that experiment participants exhibited overlapping values in the range of motion in terms of displacement, velocity and acceleration (see Fig. 3). Similarly, we did not find significant changes in performance from Day-1 to Day-3 for the joint entropy (Mean:-0.012, CI:-0.35,0.38), covariance of displacement (Mean:-2.3e-4, CI:-7.9e-4,1.2e-3), velocity (Mean:-1.2e-3, CI:-0.013,0.015), or acceleration (Mean:-0.035, CI:-0.34,0.41).

While overall group changes were not significant, some *individuals* did in fact exhibit significant increases or decreases in range of motion. In terms of displacement, we observed some agreement between consecutive days for an individual’s displacement data (Between Day-1 and Day-2, *R*^*2*^ = 0.55; between Day-2 and Day-3: *R*^*2*^ = 0.57). However, the agreement between Day-1 and Day-3 was poor (*R*^*2*^ = 0.05), indicating that participants could no longer be described by their initial evaluation. We found that some individuals did in fact significantly change in range, with both decreases and increases. As shown in Fig. 3, individuals who exhibit non-overlapping error bars (95 % CI) demonstrate a significant change across days. Similar results were found for velocity and acceleration.

We performed a similar classification analysis on the histogram data for the single sessions from 10 healthy individuals. Interestingly, healthy individuals were not as easy to discriminate as stroke survivors. While there was the same trend of less confusion for higher derivatives, the percentage of correct classification for each trial block was 56.2 %, 30.0 %, and 11.2 %, for acceleration, velocity, and displacement, respectively. The overall sensitivity of correct identification of an individual’s data as a whole was 0.97 for acceleration, 0.81 for velocity, and 0.62 for displacement.

To determine whether the identified features persisted across days, we further examined whether training data from each day could identify subsequent days. Focusing on acceleration data, we found similar classification success for consecutive days as same day analysis (Day-1 to Day-2 identification of single trials: 48.8 %; Sensitivity: 0.98) and poorer results for training and test data separated by two days (Day-1 to Day-3 identification of single trials: 33.8 % for acceleration; Sensitivity: 0.88 % for acceleration). Similar results were found for displacement and velocity (see Table 3).

**Table 3:** Classification accuracy (%) and sensitivity (*S*) of trial identification for data within and between days

Displacement			
	Day-1	Day-2	Day-3
Day-1	41.2 %, 0.88	46.5 %, 0.94	35.0 %, 0.88
Day-2		42.5 %, 0.93	36.3 %, 0.91
Day-3			46.2 %, 0.97
Velocity			
Day-1	51.2 %, 0.96	46.5 %, 0.94	32.5 %, 0.82
Day-2		68.8 %, 0.97	41.2 %, 0.92
Day-3			61.3 %, 0.96
Acceleration			
Day-1	75.0 %, 0.98	48.8 %, 0.98	33.8 %, 0.88
Day-2		86.2 %, 1.00	70.0 %, 0.98
Day-3			77.5 %, 1.00

## Discussion

This study investigated how the distributions of self-directed motor exploration can reveal patterns of abnormal coordination, and how these patterns can change with training. While the more traditional summary metrics we chose failed to show a common effect of training, our analysis of movement distribution revealed that more modeling components were needed to accurately fit the observed data from stroke survivors after training. A second finding was that patients were distinct from each other, in terms of classification features found from movement distributions. While no statistical summary can ever completely describe an individual’s capabilities, the methods introduced in this study could provide a new approach to both concisely summarize and detail a person’s movement tendencies.

Our examination of model components provides evidence of changes in motor capability due to practice. Qualitatively, the movement distributions of stroke survivors were more complex than those of age-matched control participants. For healthy individuals, overall range of motion was typically much larger, while fewer components were necessary to describe their distributions of acceleration. These features of movement distributions for the aged-matched control group indicates that a larger number of model components does not necessarily imply greater capability. Interestingly, the effect of extended practiced over the course of multiple training sessions appeared to promote even greater complexity in movement distributions for stroke survivors. It might be supposed that the increased complexity was a result of fatigue that intensified the existing abnormal movement tendencies. For example, fatigue could manifest as even greater under-expression of certain regions of the state-space. However, visual inspection of the distributions of acceleration in some cases revealed the emergence of new expanses of histogram data that appeared on subsequent training days (see Fig. 2). Consequently, a more plausible explanation for needing additional model components is that motor exploration allowed stroke survivors to recover some movement expressions that were missing from the typical distribution of acceleration. In a study with stroke survivors performing goal directed movements, Mukherjee at al. (2013) found that entropy increased with training, indicating reduction of signal repetitiveness within each movement [31]. Similarly, the increased in the number of components suggests an expansion to more movement capabilities during free motor exploration.

**Fig. 6:**
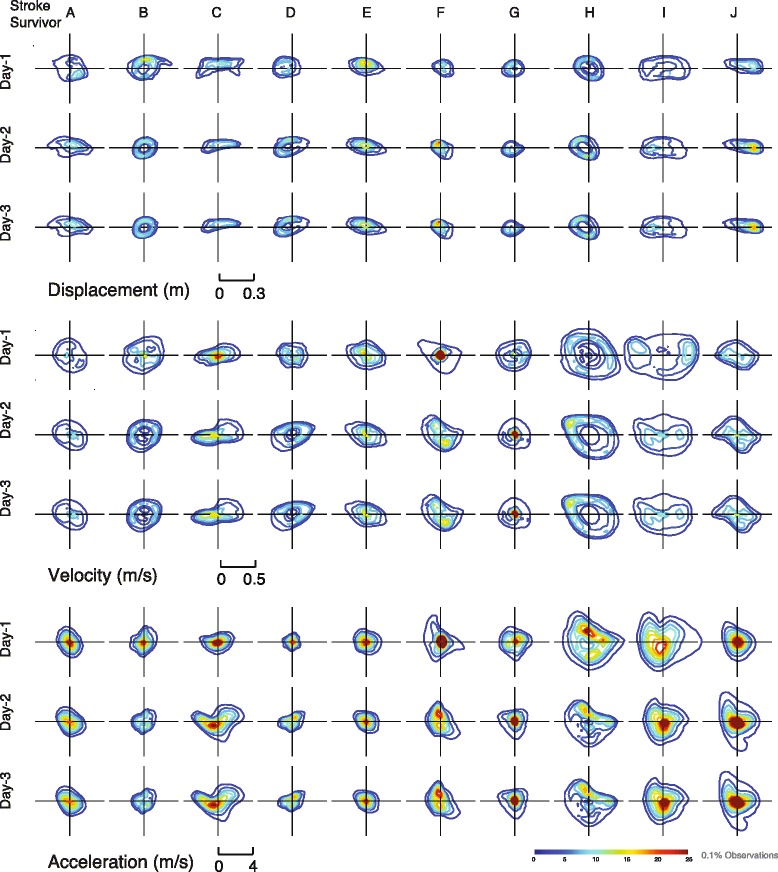
Individual Distribution plots. Contour plots for the individual histograms for displacement, velocity, and acceleration for all 10 stroke survivors (by column) for three training days

The approach in this study of examining movement trends in terms of model distributions is motivated by a need to characterize the highly variable nature of self-directed practice. While it is difficult to determine whether the individual observed deficits in such distributions reflect intrinsic motor impairments rather than volitional choice (perhaps arising from mood, inattention, etc.), we speculate that velocity and acceleration distributions are less influenced by conscious control or manipulation. Furthermore, as more data is collected we expect the systematic effects of motor impairments to dominate over the less repeatable influences from choice. Note that some individuals in this study exhibited highly cyclic patterns of movement (see Fig. 6), which were likely influenced by the person’s particular goals-directed tasks in other parts of the experimental protocol. However, the impact of this behavior is markedly less apparent in the domain of acceleration. More study is needed to determine how instructions affect these distribution outcomes, and how impairment can be isolated from other effects.

While our results have revealed evidence of individual differences and changes in the patterns of motor exploration, it is still worth asking how such analysis can relate to functional capability. Our results showed that our chosen scalar metrics did not reveal significant changes across days, nor for the most part did they reveal correlations between motor exploration and goal-directed movement. We argue, however, that most traditional scalar metrics inherently fail to capture the true statistical and multi-dimensional aspects of motor exploration data. Distribution analysis, on the other hand, reveals general statistical properties of kinematic variables that must then have consequences on a variety of tasks. The fact that the covariance of acceleration was significantly correlated with several metrics in circular movement could indicate that *descriptions of distribution* have relatively more utility. We expect that individuals that are able to express a wider range of movement states should have more resources available for general coordination.

While distribution analysis of motor exploration could offer a powerful description of movement patterns, the relationships of these patterns to functional skill must be investigated further. It is plausible that differences in distribution features across individuals could be related to clinical assessments such as the Fugl-Meyer test, or even the lesion location. Researchers have explored various notions of “motor primitives”, either in terms of oscillators or field functions [45–47], which act as fundamental building blocks of goal-directed actions. Similarly, our analysis of the increasing model components is suggestive of building elements of movement ability. Further study is needed to determine if extended training might result in more normally distributed acceleration, such as those exhibited by healthy individuals in this study. We expect that the distribution model components would likely reveal more predictive power in the sections of clinical scores related to gross arm coordination.

Classification of histogram data showed that there was poorer accuracy in identifying healthy individuals compared to stroke survivors, which suggests that motor deficits contribute significantly to the uniqueness of motor exploration patterns. The accuracy in classification we found in this study would not be possible with summary metrics, such as simple range of motion or maximum speed. While it is perhaps unsurprising that individuals exhibit unique signatures, we found that the healthy distribution patterns were simpler and more difficult to distinguish compared to stroke. Healthy individuals may have less distinct features than stroke survivors, because they may be more willing to fully explore. Our examination of distributions might then reveal insight into motor deficits by identifying how movement gestures differ those of the normal population. While our chosen scalar metrics did not sufficiently distinguish between individuals, it is worth noting that participants exhibited evident differences in how they changed across days. In some cases, participants even exhibited a decrease in displacement range (see Fig. 2). We note, however, that the stroke survivors considered in this analysis did not receive any training forces or other forms of novel intervention. It is possible that the lack of feedback for knowledge of results allowed participants to choose their own learning objectives. Consequently, maximal limits of performance extent may have deprioritized.

Interestingly, we found clearer separation between individuals in the distributions of acceleration compared to the lower derivatives (displacement and velocity). This trend was especially apparent from the results of classification accuracy (see Fig. 5), and was true for both stroke survivors and age-matched controls. We observed that *displacement* distributions were generally more uniform, and hence more similar across individuals. It is also possible that acceleration is more closely connected to force production or motor planning, which has been posited to be a source of motor deficits in stroke [8, 48–50]. Loss of coordination, weakness, and abnormal reflex patterns, manifest from a loss of neural resources needed to send motor commands. Consequently, differences in such control might be more evident in patterns of how muscles apply force.

The finding of distinct movement patterns has implications for improving assessment methods of motor impairment. The distribution analysis employed in this study might provide new approaches for identifying underlying causes of motor deficits and for devising more focused treatment. Characterizing motor deficits is a daunting task in part because of the wide variety of pathologies. Current methods in robot-based assessment focus largely on discrete movements (such as a reach to a target) for measuring performance, but larger datasets from exploratory movement could enable a more complete description of capability. For example, the distribution of data at certain states could exhibit sparseness that suggests a lack of expression. Limits in range of motion should also easily identify sharp changes in distribution that are consistent with hard biomechanical limits such as the changes arm mechanics due to contracture.

Beyond describing motor impairment, the distribution analysis in this study could provide powerful tools for designing customized therapy. The differences between stroke survivors have been well recognized, especially amongst clinicians. Treatment would be greatly simplified by finding a universal approach for rehabilitation that is effective for a large number of individuals. However, a framework for characterizing individual differences offers a tremendous opportunity—enhancing the impact of a given intervention by focusing on an individual’s particular training needs. The rich information available in movement distribution analysis has the potential to enhance customization in a way not possible with traditional measurements. Recent work has shown how interactive machines can inform a direct mathematical relationship between movement deficits and applied interventions [51]. In a similar fashion, the distribution analysis in this study could be used as a mathematical framework to direct practice patterns towards neglected movements. It is at this point unclear how the observed deficits in movement distributions can be attributed to underlying impairments. Nonetheless, one plausible strategy is to employ the nervous system’s natural use-dependent learning mechanisms [52] to shift these tendencies *away* from unwanted patterns. Research in *constraint-induced therapy* [53, 54] has been shown to help reverse learned non-use [55] of the affected limb. Similarly, deficits in the space of movement gestures might be similarly affected by re-shaping frequency of usage.

## Conclusions

Distribution analysis revealed individual patterns of abnormal coordination and changes in these patterns with training. These findings were not apparent from scalar metrics that simply summarized properties of motor exploration. Our results showed that more modeling components were needed to accurately fit the observed data from stroke survivors after training. In addition, classification using linear discriminant analysis revealed that movement patterns were identifiable by individual. We also provided a case study of healthy participants, revealing distributions generally simple in structure and more difficult to distinguish between individuals. Because of its ability to identify individual-specific patterns of abnormal coordination, the distribution analysis approach in this study could provide powerful tools for designing customized therapy.

## Abbreviations

CI: Confidence interval
Coeff: Coefficient
M: Meters
s: Seconds
SD: Standard deviation
UEFM: Upper extremity Fugl-meyer
